# Causes of congenital corneal opacities and their management in a tertiary care center

**DOI:** 10.5935/0004-2749.20200023

**Published:** 2020

**Authors:** Remzi Karadag, Christopher J. Rapuano, Kristin M. Hammersmith, Parveen K. Nagra

**Affiliations:** 1 Department of Ophthalmology, School of Medicine, Istanbul Medeniyet University, Istanbul, Turkey; 2 Cornea Service, Wills Eye Hospital, Sidney Kimmel Medical College at Thomas Jefferson University, Philadelphia, USA

**Keywords:** Cornea/abnormalites, Corneal opacity/congenital, Keratoplasty, penetrating, Peters anomaly, Tertiary healthcare, Córnea/anormalidades, Opacidade da córnea/ con gênito, Ceratoplastia penetrante, Atenção terciária à saúde

## Abstract

**Purpose:**

To evaluate causes and management of congenital corneal opacities (CCO) diagnosed in a tertiary care eye center and to compare the data with a previous study at the same institution.

**Methods:**

Computerized medical records in all patients with congenital corneal opacities diagnosed in the Cornea Service at Wills Eye Hospital (Philadelphia, PA) between January 1, 2007, and December 31, 2015, were retrospectively reviewed. Children aged 12 years and younger at the first visit were included in the study. Patients’ demographics, ocular diagnosis, laterality, associated ocular abnormalities, other ocular surgery performed prior or subsequent to the first visit, and their treatment were extracted from the medical records.

**Results:**

A total of 77 eyes in 56 patients were examined. The mean age at presentation was 32.8 ± 44.2 months, with the mean follow-up period of 26.7 ± 30.1 months. The most frequent diagnosis was Peters anomaly (53.2%), followed by limbal dermoid (13.0%), aniridia with glaucoma and microphthalmos (6.5%), sclerocornea and congenital glaucoma (5.2%), idiopathic (3.9%), Axenfeld-Rieger anomaly and Hurler syndrome (2.6%), and microcornea (1.3%). Primary keratoplasty was performed in 26 eyes, with the outcome rate in the clear cornea of 76.0% during the follow-up.

**Conclusion:**

Peters anomaly is the most common cause of congenital corneal opacities encountered at our institution. Penetrating keratoplasty is the most frequent choice of corneal surgery to treat congenital corneal opacities. Additional interventions during penetrating keratoplasty were moderately positively correlated with graft failure. This study also shows the rates of some etiologies of that changed over the recent decades in our tertiary care Cornea Service. Although Peters anomaly remains the most common presenting reason for congenital corneal opacities, its rate appears to be increasing over the recent decade. Congenital corneal opacities due to birth trauma, which is one of the preventable causes, were observed in a previous study in our clinic; however, no new cases were noted in this study.

## Abstract (pt)

**Objetivo:**

Avaliar as causas e o controle das opacidades corneanas congênitas diagnosticadas em um centro oftalmológico de atendimento terciário e comparar os dados com um estudo anterior realizado na mesma instituição.

**Métodos:**

Prontuários médicos informatizados de todos os pacientes com opacidade corneana congênita diagnosticada no Serviço de Córnea no Wills Eye Hospital (Filadélfia, PA) entre 1º de janeiro de 2007 e 31 de dezembro de 2015 foram revisados retrospectivamente. Crianças com 12 anos ou menos na primeira consulta foram incluídas no estudo. A demografia dos pacientes, o diagnóstico ocular, a lateralidade, as anormalidades oculares associadas, outras cirurgias oculares realizadas antes ou após a primeira consulta e o tratamento foram extraídos dos prontuários médicos.

**Resultados:**

Um total de 77 olhos de 56 pacientes foi examinado. A idade média de apresentação foi de 32,8 ± 44,2 meses, com um tempo médio de acompanhamento de 26,7 ± 30,1 meses. O diagnóstico mais frequente foi anomalia de Peters (53,2%), seguido por dermóide límbico (13,0%), aniridia com glaucoma e microftalmia (6,5%), esclerocórnea e glaucoma congênito (5,2%), idiopático (3,9%), síndrome de Axenfeld-Rieger e síndrome de Hurler (2,6%) e microcórnea (1,3%). Ceratoplastia primária foi realizada em 26 olhos, com desfecho de córnea clara de 76,0% durante o acompanhamento.

**Conclusão:**

A anomalia de Peters é a causa mais comum de opacidade corneana congênita encontrada em nossa instituição. A ceratoplastia penetrante é a escolha mais frequente de cirurgia corneana para o tratamento de opacidades corneanas congênitas. Intervenções adicionais durante a ceratoplastia penetrante foram moderadamente correlacionadas positivamente com a falha do enxerto. Este estudo também mostra as taxas de algumas etiologias do que mudou ao longo faz últimas décadas em nosso serviço de córnea de atendimento terciário. Embora a anomalia de Peters continue a ser a causa mais comum das opacidades congênitas da córnea, sua taxa parece estar aumentando na última década. Opacidades congênitas da córnea devido a trauma no nascimento, que é uma das causas evitáveis, foram observadas em um estudo anterior em nossa clínica; no entanto, nenhum caso novo foi observado neste estudo.

## INTRODUCTION

Congenital corneal opacities (CCO) are defined as a group of diseases characterized by loss of transparency in the corneal tissue at birth or during the first 4 weeks of life^(1,2)^. Etiologic factors affecting the anterior segment differentiation between the 6th and 16th weeks of gestation are responsible for most CCOs. These factors may be infectious, genetic, metabolic, developmental, traumatic, toxic, idiopathic, or a combination of these^(1,2)^. Most cases are bilateral and are often observed with other ocular malformations. They may also be accompanied by complex systemic disorders. CCO incidence is reported from 2.2 to 3.11 per 100,000 births^(3-5)^. Although CCO is a rare entity, prompt diagnosis and treatment are very important due to the high risk of amblyopia and possibility of lifelong visual impairment in bilateral cases^(3,4)^. Its diagnosis, treatment, and follow-up were associated with their own set of difficulties^(1-4)^.

This study aimed to analyze the data (patients’ demographics, ocular diagnosis, laterality, associated ocular abnormalities, other ocular surgery performed prior or subsequent to the first visit, and the treatment) of CCO children diagnosed in the Cornea Service at Wills Eye Hospital between January 2007 and December 2015 and compare the results with another study also conducted in our Service a decade prior as well as other studies in the literature.

## METHODS

Computerized medical records of all patients with CCO diagnosed in the Cornea Service at Wills Eye Hospital (Philadelphia, PA) between January 1, 2007, and December 31, 2015, were retrospectively reviewed. Patients were identified through a computerized diagnosis code search. Children aged 12 years and younger at their first visit to our Cornea Service were included in the study. Patients with eyes that underwent previous corneal surgery in another hospital were excluded. Patients’ de mographics, ocular diagnosis, laterality, associated ocular abnormalities, other ocular surgeries performed prior or subsequent to the first visit, and the treatment and surgical outcomes were extracted from medical records. This study protocol was approved by the Wills Eye Hospital Institutional Review Board and conducted in accordance with the Declaration of Helsinki.

The SPSS software version 16 (SPSS Inc., Chicago, IL, USA) were used for statistical analyses. The Kolmogorov-Smirnov test was used to investigate variables in order to determine whether or not they are normally distributed. Descriptive statistical methods (mean, standard deviation, median, frequency, ratio, minimum, and maximum) were used to evaluate this study’s data. The Spearman’s correlation test was used to evaluate the data. P-values of <0.05 were considered statistically significant.

## RESULTS

A total of 96 eyes in 64 patients (32 unilateral, 32 bilateral) with CCO were identified from the medical records. After excluding 19 eyes that had corneal surgery prior to the first visit at Wills, 77 eyes in 56 patients were included in the analysis. Among them, 30 (53.6%) were females and 26 (46.4%) were males. Both eyes were diagnosed with CCO in 21 patients (38%). The mean age at presentation was 32.9 ± 44.2 (range, 0.2-144) months, with the mean follow-up period of 26.7 ± 30.1 (range 0.1-103) months. The most frequent diagnosis was Peters anomaly (41 eyes, 53.2%), followed by limbal dermoid (13.0%), aniridia with glaucoma (6.5%), microphthalmos (6.5%), sclerocornea (5.2%), congenital glaucoma (5.2%), idiopathic corneal opacity (3.9%), Axenfeld-Rieger anomaly (2.6%), Hurler syndrome (2.6%), and microcornea (1.3%) (Table 1). Corneal findings in all eyes at presentation are presented in table 2. Corneal findings in eyes with Peters anomaly are shown in table 3. The most common findings were central haze/ scar (27 eyes, 65.9%), paracentral haze/scar (12.2%), edema (9.7%), and pannus (9.7%). Thirty eyes (39.0%) underwent corneal surgery, which included penetrating keratoplasty in 25 eyes (32.5%), limbal dermoid excision in 3 (3.9%), keratoprosthesis in 1 (1.3%), and lamellar keratectomy in 1 (1.3%) (Table 4).

**Table 1:** Diagnosis for congenital corneal opacity

Diagnosis	Number of eyes	%
Peters anomaly	41	53.2
Limbal dermoid	10	13.0
Aniridia with glaucoma	5	6.5
Microphthalmos	5	6.5
Sclerocornea	4	5.2
Congenital glaucoma	4	5.2
Idiopathic corneal opacity	3	3.9
Axenfeld-Rieger Anomaly	2	2.6
Hurler syndrome	2	2.6
Microcornea	1	1.3

**Table 2:** Presenting timely corneal findings

Corneal findings	Number of eyes	%
Central haze/scar	37	48,1
Diffuse full thickness haze	13	16.9
Corneal mass	9	11.7
Neovascularization/pannus	11	14.3
Paracentral/peripheral haze/scar	12	15.6
Edema	8	10.4
Microcornea	5	6.5
Band keratopathy	4	5.2
Scleralization	4	5.2
Buphthalmos	5	6.5
Posterior embryotoxon	5	6.5
Posterior keratoconus	1	1.3

* More than one finding may occur in the same eye.

**Table 3:** Corneal findings in 41 eyes with Peters anomaly

Corneal findings	Number of eyes (n)	%
Central haze/scar	27	65.9
Paracentral haze/scar	5	12.2
Edema	4	9.7
Pannus	4	9.7
Posterior embryotoxon	3	7.3
Band keratopathy	3	7.3
Neovascularization	3	7.3
Diffuse full thickness haze	3	7.3
Peripheral haze/scar	2	4.9
Indistinct limbus	2	4.9
Microcornea	1	2.4
Posterior keratoconus	1	2.4

* More than one procedure may occur in the same eye.

**Table 4:** Surgeries performed prior to presentation, corneal surgeries performed at our institution, and concurrent and subsequent surgeries to the corneal surgeries.

	Number of eyes^*^	%
Prior to time of presentation		
Glaucoma drainage device	4	5.2
Trabeculectomy	4	5.2
Trabeculotomy/goniotomy	2	2.6
Cataract extraction	4	5.2
Phacoemulsification with lens implant	3	3.9
Cyclophotocoagulation	3	3.6
Strabismus surgery	2	2.6
Corneal surgery		
Penetrating keratoplasty	25	32.5
Lamellar keratectomy	1	1.3
Primary keratoprosthesis	1	1.3
Limbal dermoid excision	3	3.9
Second penetrating keratoplasty	5	6.5
Keratoprosthesis after penetrating keratoplasty	1	1.3
Third penetrating keratoplasty	2	2.6
Fourth penetrating keratoplasty	1	1.3
Concurrent surgery to corneal surgery		
Lensectomy	7	9.1
Anterior vitrectomy	7	9.1
Peripheral iridectomy	12	15.6
Release of synechiae	7	9.1
Cyclophotocoagulation	2	2.6
Amniotic membrane transplantation	2	2.6
Pars plana vitrectomy	1	1.3
Laser retinopexy	1	1.3
Subsequent or additional surgery to corneal surgery		
Glaucoma drainage device	2	2.6
Cataract extraction	2	2.6
Strabismus surgery	3	3.9
Pupilloplasty	2	2.6
Phacoemulsification with lens implant	2	2.6
Revision of glaucoma drainage device	1	1.3

* More than one procedure may occur in the same eye.

Patients’ diagnoses are summarized in table 5.

**Table 5:** Diagnosing eyes undergoing primary penetrating keratoplasty

Diagnosis	Eyes (n)	%
Peters anomaly	20	76.9
Aniridia glaucoma	2	7.7
Sclerocornea	2	7.7
Congenital glaucoma	1	3.8
Idiopathic corneal opacity at birth	1	3.8

Ocular surgery, other than corneal surgery, was performed in 16 eyes prior to the first visit at Wills. Concurrent surgeries were performed in 15 eyes at the time of corneal surgery, as shown in table 4. Five of 25 eyes that underwent penetrating keratoplasty underwent a second keratoplasty, and one eye underwent keratoprosthesis (four of these six eyes had Peters anomaly and the other two eyes had sclerocornea) (24% graft failure). Keratoplasties were performed in three eyes, and a fourth keratoplasty in one eye. When analyzing six eyes with graft failure, four had Peters anomaly (20 total eyes had Peters anomaly) and two of them had sclerocornea (2 total eyes had sclerocornea). The graft failure rate was 20% in patients with Peters anomaly and 100% in those with sclerocornea.

Glaucoma was observed in 24 eyes (31.2%) prior to their first visit or during our follow-up at Wills. Glaucoma surgeries were performed 13 eyes prior to their first visit including four glaucoma drainage devices, four trabeculectomies, 3 cyclophotocoagulations, and two trabeculotomy/goniotomy procedures. Concurrent glaucoma surgeries were performed in two eyes (cyclophotocoagulation) at the time of corneal surgery. Additionally, subsequent glaucoma surgeries were performed in two eyes, and revision of a glaucoma drainage device was performed in one eye after a corneal surgery (Table 4). Seven of the 24 eyes were followed with medical glaucoma treatment. The correlation between the presence of glaucoma and history of previous surgery before the presentation was statistically significantly moderately positive (r=0.498, p=0.010). Concurrent intervention at the time of primary keratoplasty was statistically moderately correlated with the need for secondary keratoplasty (r=0.445, p=0.026). The outcome rate in the clear cornea during the follow-up was 76.0%.

## DISCUSSION

Patients with CCO are highly at risk of deprivation amblyopia since a clear cornea is required for healthy vision development. Furthermore, because these conditions are commonly bilateral, patients are highly at risk for lifelong visual impairment. Therefore, diagnosis, determination of concomitant ocular and systemic pathologies, and treatment of CCO as early as possible are essential^(1,2,5)^.

CCO is an etiologically heterogenic situation. Its occurrence CCO vary depending on the developmental level of the country and genetic and environmental factors. Only a few studies investigated these conditions^(1-5)^. However, most of the information in these studies was obtained from pediatric keratoplasty. Not surprisingly, the most common reasons for CCO vary between different countries^(1-8)^.

Peters anomaly is found in 40.3% and 72.7% of patients in two of the largest studies on CCO, the first one in the USA^(1)^, in the previous study in our institution, and the second one in Japan^(2)^. Sclerocornea is the second most common etiology, with the occurrence rate of 18% in a US study^(1)^ and anterior staphyloma in a Japanese study^(2)^. In the present study, Peters anomaly (53.2%) was the most common etiology of CCO, followed by limbal dermoid (13.0%), aniridia with glaucoma and mi crophthalmos (6.5%), sclerocornea and congenital glaucoma (5.2%), idiopathic (3.9%), and Axenfeld-Rieger anomaly and Hurler syndrome (2.6%) according to frequency. This study also shows CCO rates that changed over the recent decades in our tertiary care Cornea Service. In our previous study, the most common primary cause of CCO was Peters anomaly (40.3%), followed by sclerocornea (18.1%), dermoid (15.3%), congenital glaucoma and idiopathic (6.9%), microphthalmia (4.2%), and birth trauma and metabolic diseases (2.8%). Even though Peters anomaly remains the most common etiology of CCO, its rate appears to be increasing in the recent decade. CCO due to birth trauma, which is one of the preventable causes, was observed in 2.8% of patients in our clinic’s previous study^(1)^; however, no new case was detected in this study. Although idiopathic CCO was observed in 6.9% of the patients in our clinic’s previous study^(1)^, its rate appears to be decreasing in the recent decade (3.9%). Bilateral involvement of CCO was observed in 73.6% of patients from the Far East,^^2^^ 55.3% in our clinic’s previous study^(1)^, and 54.6% in this study.

In a Saudi Arabian study, Al-Ghamdi et al. reported 130 patients with primary pediatric keratoplasty had CCO. In their cases, the most common etiologies were congenital glaucoma (33.7%), congenital hereditary endothelial dystrophy (CHED) (26.9%), and Peters anomaly (21.5%)^(6)^. In Patel et al.’s study in New Zealand, nine pediatric keratoplasties were performed over a 13-year period because of CCO, and 44.7% of them had Peters anomaly, followed by CHED (22.2%), idiopathic (22.2%), and limbal dermoid (11.1%)^(7)^. In a Finland study, Majander et al. evaluated 14 eyes of seven patients with CCO who underwent anterior segment optic coherence tomography. Peters anomaly with the rate of 71.4%, congenital corneal staphyloma with 21.4%, and microphthalmos with 7.1% were diagnosed in these eyes^(8)^.

Because the risk of amblyopia and visual impairment is high in eyes with CCO, keratoplasty, most frequently penetrating keratoplasty, is the most common treatment method^(7-10)^. Penetrating keratoplasty is an especially risky procedure in children because their ocular tissues continuously grow, scleral flexibility is high, follow up is more difficult, and other ocular pathologies are commonly present in this age group^(8,9)^. In our study, primary penetrating keratoplasty was the most common procedure with a rate of 32.5%, which was similar with our clinic’s previous study (33.1%)^(1)^.

In this study, among the patients who underwent primary penetrating keratoplasty due to CCO, 76.9% had Peters anomaly, followed by 7.7% with glaucoma and 7.7% with sclerocornea. Central corneal opacity was the most common finding with a rate of 78.1% in eyes with Peters anomaly. The full thickness of the central corneal pathology results in higher rates of penetrating keratoplasty in these patients as compared to other diseases. In studies of pediatric penetrating keratoplasty, where Peters anomaly was observed in majority of patients, clear graft at the final visit was found in 29-75.1%^(6-13)^. Clear grafts were noted in 66.7% in the previous study (mean follow-up time, 33.1 months) from our clinic^(1)^ and 76.0% at the last visit in this study (mean follow-up time, 26.7 months). When analyzing 6 eyes with graft failure, 4 had Peters anomaly (20 in total) and two had sclerocornea (2 in total). The graft failure rate was 20% in patients with Peters anomaly and 100% in those with sclerocornea. Previous studies showed that graft failure rates were also higher in sclerocornea as compared to other CCO etiologies^(1,13,14)^. While sclerocornea was previously considered a separate and distinct diagnosis, several pediatric ophthalmologists now consider it as an (often severe) manifestation of various other conditions, most commonly Peters anomaly and Axenfeld-Rieger anomaly^(15)^.

In this study, additional interventions during PK were moderately positively correlated with graft failure. In other studies in the literature, complicated cases requiring additional interventions also had worse prognosis^(9,12)^. Glaucoma was the most common preand pos toperative complicating factors^(1,9,10,12)^. Glaucoma history was also moderately positively correlated with his tory of previous surgery before performing primary ker atoplasty in this study. Since glaucoma control is critically important for graft survival and to maintain vision, the more eye surgeries performed, the poorer the long-term prognosis is for the graft.

In conclusion, although Peters anomaly remains the most common presenting etiology for CCO, its rate appears to be increasing in the recent decade. CCO due to birth trauma, which is one of the preventable causes, was observed in our clinic’s previous study; however, no new cases were observed in this study. Although idiopathic CCO was observed in our clinic’s previous study, its rate appears to be decreasing in the recent decade. Although CCO is rare in most clinical practice, early diagnosis and management is deemed necessary to decrease the risk of lifelong visual problems. However, we believe that knowing the success rates of keratoplasty for CCO is also important so that patients and their caregivers can be prepared to face this condition by understanding the usual complications and possible re-operations to realize their expectations about the final visual outcomes and their quality of life in the future.
